# Progress in Research on the Role of the Thioredoxin System in Chemical Nerve Injury

**DOI:** 10.3390/toxics12070510

**Published:** 2024-07-15

**Authors:** Xinwei Xu, Lan Zhang, Yuyun He, Cong Qi, Fang Li

**Affiliations:** 1 School of Medicine, Jiangsu University, Zhenjiang 212013, China; 2212213084@stmail.ujs.edu.cn (X.X.); 19805662010@163.com (L.Z.); 19802596761@163.com (Y.H.); 2 Department of Pharmacy, Jurong People’s Hospital, Jurong 212400, China; wsqca@163.com

**Keywords:** chemical nerve injury, Trx system, Trx reductase, Trx-interacting protein

## Abstract

(1) Background: Various factors, such as oxidative stress, mitochondrial dysfunction, tumors, inflammation, trauma, immune disorders, and neuronal toxicity, can cause nerve damage. Chemical nerve injury, which results from exposure to toxic chemicals, has garnered increasing research attention. The thioredoxin (Trx) system, comprising Trx, Trx reductase, nicotinamide adenine dinucleotide phosphate, and Trx-interacting protein (TXNIP; endogenous Trx inhibitor), helps maintain redox homeostasis in the central nervous system. The dysregulation of this system can cause dementia, cognitive impairment, nerve conduction disorders, movement disorders, and other neurological disorders. Thus, maintaining Trx system homeostasis is crucial for preventing or treating nerve damage. (2) Objective: In this review study, we explored factors influencing the homeostasis of the Trx system and the involvement of its homeostatic imbalance in chemical nerve injury. In addition, we investigated the therapeutic potential of the Trx system-targeting active substances against chemical nerve injury. (3) Conclusions: Chemicals such as morphine, metals, and methylglyoxal interfere with the activity of TXNIP, Trx, and Trx reductase, disrupting Trx system homeostasis by affecting the phosphatidylinositol-3-kinase/protein kinase B, extracellular signal-regulated kinase, and apoptotic signaling-regulated kinase 1/p38 mitogen-activated protein kinase pathways, thereby leading to neurological disorders. Active substances such as resveratrol and lysergic acid sulfide mitigate the symptoms of chemical nerve injury by regulating the Ras/Raf1/extracellular signal-regulated kinase pathway and the miR-146a-5p/TXNIP axis. This study may guide the development of Trx-targeting modulators for treating neurological disorders and chemical nerve injuries.

## 1. Introduction

Nerve damage results from the disruption of nerve structure integrity and the subsequent impairment of nerve function. Tumors, inflammation, trauma, and immune disorders can damage the nervous system, leading to dementia, cognitive impairment, nerve conduction disorder, movement disorder, and nutritional disorder in the damaged area. In addition, nerve damage—central or peripheral—can lead to various disorders such as Parkinson’s disease (PD), diabetic peripheral neuropathy (DPN), Alzheimer’s disease (AD), and autism spectrum disorder [1]. This shows that nerve damage iswidespread. As per the 2015 Global Disease Study, neurological disorders are the second leading cause of mortality, accounting for 16.8% of all deaths worldwide. Over the past 25 years, a 36% increase has been noted in the rate of mortality from neurological disorders [2].

Chemical nerve injury refers to toxic chemical-induced nerve damage. Exposure—through contact, inhalation, accidental ingestion, or environmental exposure to severe pollution—to these chemicals (e.g., metals) damages the nervous system (in both humans and animals) by disrupting normal nerve conduction. Owing to the ubiquity of chemical substances, particularly neurotoxic chemicals, increasing research attention has been paid to chemical nerve injury.

Recent evidence implicates the Trx system in the maintenance of redox homeostasis in the central and peripheral nervous systems, while its results are altered in PD [3], DPN [4], and AD [5]. Genetic and environmental factors (nutrition, metals, and toxins) are known to impact the function of the Trx system, thereby contributing to neuropsychiatric disease [6]. Chemicals such as morphine, metals, and methylglyoxal can disrupt the homeostasis of the Trx system, resulting in nerve damage. Timely regulation of the Trx system can prevent chemical nerve injury. Therefore, the concept of targeting this system for treating nerve damage has gained increased attention in the research community. Thus, we conducted this review to identify the factors influencing the homeostasis of the Trx system, assess the involvement of its homeostatic imbalance in chemical nerve injury, and investigate the therapeutic potential of Trx system-targeting active substances against chemical nerve injury.

## 2. Trx System

The Trx system primarily comprises different isoforms of Trx and Trx-like proteins, isoforms of Trx reductase (TrxR), nicotinamide adenine dinucleotide phosphate (NADPH; reduced coenzyme II or triphosphopyridine nucleotide), and several Trx-interacting proteins, including TXNIP and apoptotic signaling-regulated kinase 1 (ASK1). This system is a widely distributed in vivo NADPH-dependent disulfide reductase system. It is a major defense mechanism against oxidative stress and apoptosis. Enhanced TrxR activity alleviates intracellular oxidative stress and protects cellular functions [7], thereby maintaining redox homeostasis in the central nervous system. Under oxidative stress and in inflammatory conditions, nerve cells release Trx into the extracellular compartment, where Trx exerts cytoprotective effects [8]. TXNIP binds Trx within the cell. Notably, elevated levels of TXNIP result in the direct inhibition of Trx expression.

### 2.1. Trx

**Figure 1:**
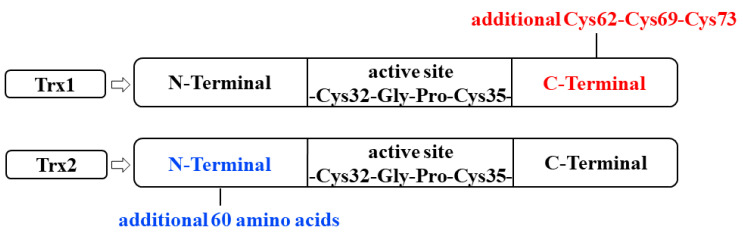
Structure of Trx. The active center of Trx is “-Cys32-Gly-Pro-Cys35-”. Trx1 has 3 additional cysteine residues at the carboxyl terminus; Trx2 has an additional 60-amino acid sequence at the amino terminus, this signal guides the transfer of Trx2 to the mitochondria. Trx: thioredoxin; Cys: cysteine residues.

Trx is an oxidoreductase essential for oxidized proteins to recover to their reduced active form. It possesses antioxidative, pro-cell growth, protein-binding, and antiapoptotic properties. To date, Trx has been described as a cytokine for which many cell-activating effects have been observed in the micromolar range and whose cellular effects require adjuvants for activation [9]. Trx was first isolated from *Escherichia coli* in 1964 by Laurent et al. [10]. With technological advancements, Trx has been detected in many prokaryotic and eukaryotic organisms. In mammalian cells, it is categorized into Trx1, Trx2, and Trx3 (specific to spermatogonial cells). Trx1 is found in the cytoplasm and nucleus, whereas Trx2 is found exclusively in the mitochondria. Trx1 and Trx2 are the predominant isoforms of Trx in humans. Both isoforms feature two cysteines in their active sites. However, only Trx1 has three additional cysteine residues at the carboxyl terminus; these residues (-Cys62-Cys69-Cys73-) are involved in protein nitrosylation. By contrast, Trx2 has an additional 60-amino acid sequence at the amino terminus; this signal guides the transfer of Trx2 to the mitochondria (Figure 1) [11]. The active center of Trx (-Cys32-Gly-Pro-Cys35-) is responsible for its antioxidative properties. Any alteration in this sequence leads to conformational changes in Trx, destabilizes this biomolecule, and interferes with its reducing ability [12].

Trx dysfunction has been implicated in various diseases, including cancer, neurodegenerative disorders, and cardiovascular disease. Intravenous injection or overexpression of Trx protects against ischemic stroke injury and extends lifespan [13]. Mice overexpressing Trx exhibit reduced susceptibility to ischemia-induced brain injury. Trx regulates nerve growth factor-mediated signaling and PC12 cell growth. Consistent with this observation, Trx protects neurons (SH-SY5Y and PC12 cells) against the severe oxidative stress and injury induced by the PD-associated neurotoxin 1-methyl-4-phenylpyridinium ion (MPP^+^) [14]. Tumors exhibit elevated expression levels of Trx1. Trx1 expression is upregulated in the intestines of patients with bowel cancer. Notably, a higher expression level of Trx1 is associated with a poorer prognosis [15]. In mice lacking Trx1, a reduction was noted in the level of the Trx1–ASK1 complex in the cytoplasm, but an increase was observed in the level of ASK1 phosphorylation. These findings suggest that Trx1 knockout promotes apoptosis. Mice lacking mitochondrial Trx2 exhibit markedly reduced mitochondrial adenosine triphosphate (ATP) production, diminished electron transfer chain activity, enhanced mitochondrial apoptosis, increased reactive oxygen species (ROS) production and oxidative damage, and elevated sensitivity to apoptosis [16]. Trx is essential for mammalian development; the absence of Trx1 or Trx2 proves lethal to mouse embryos [17].

### 2.2. TrxR

**Figure 2:**
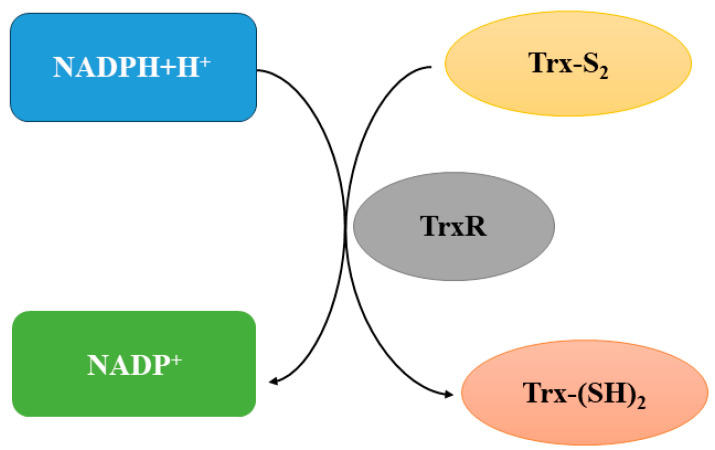
Trx system. TrxR converts the conversion of oxidized Trx or Trx-S_2_ to reduced Trx or Trx-(SH)_2_ by using electrons from NADPH. Trx: thioredoxin; TrxR: Trx reductase; NADPH: nicotinamide adenine dinucleotide phosphate.

TrxR, a major functional component of the Trx system, converts the conversion of oxidized Trx or Trx-S_2_ to reduced Trx or Trx-(SH)_2_ by using electrons from NADPH (Figure 2) [18]. Mammalian TrxR contains a selenocysteine residue in the C-terminal active site; however, low-molecular-weight prokaryotic TrxR lacks this residue. Owing to this specific nature of selenocysteine, mammalian TrxR has a distinct catalytic mechanism and a broad substrate spectrum, unlike prokaryotic TrxR. Selenocysteine is susceptible to attacks by electrophilic compounds such as 1-chloro-2,4-dinitrobenzene. In an electrophilic attack, the selenocysteine residue of mammalian TrxR alkylates with neighboring cysteine residues, which inhibits TrxR activity, and TrxR is subsequently converted into NADPH oxidase [19]. Three isoforms of TrxR can be found in the human body: TrxR1 in the cytoplasm and nucleus, TrxR2 in the mitochondria, and TrxR3 in the testicular tissue [20]. In a relevant animal study, TrxR activity persisted in the rat brain under severe selenium deficiency conditions; this observation highlights the importance of TrxR in brain function [21].

TrxR is integral to various cellular functions. It participates in all signaling pathways where Trx serves as a reducing agent. Mutations or deletions in the TrxR gene have been implicated in many neurological disorders. The expression of Trx and TrxR was found to be downregulated in the substantia nigra of a mouse model of PD and the brain tissues of deceased patients with PD. Neuronal expression of this selenoprotein is essential for the development of interneurons, which prevent seizures in addition to playing other roles [22]. TrxR is also essential for growth and development; mouse embryos lacking TrxR1 and TrxR2 exhibit abnormal growth and embryonic death on gestational days 8 and 13 [23]. TrxR reduces ribonucleotides to deoxyribonucleotides and contributes to DNA repair [24]. Furthermore, TrxR-dependent pathways maintain redox homeostasis in human-induced pluripotent stem cells and their differentiated progeny cells under varying levels of oxidative stress. Inhibition of TrxR under a low oxidative load strongly affects oxidative stress resistance [25].

### 2.3. NADPH

NADPH has been described as the universal currency for anabolic reduction reactions [26]. Through electron transfer, NADPH facilitates the TrxR-mediated reduction of Trx-S_2_ into Trx-(SH)_2_ (Figure 2). In some cases, NADPH contributes to oxidative stress by transferring electrons to molecular oxygen via the NADPH enzyme. Overstimulation of NADPH may lead to the overproduction of ROS [27].

### 2.4. TXNIP

**Figure 3:**
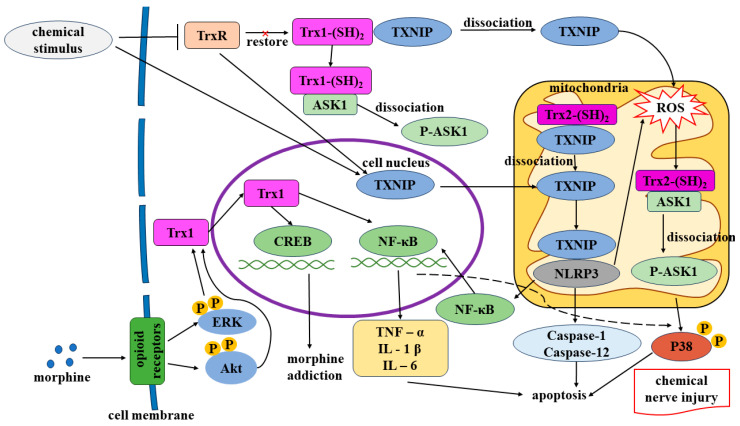
Hypothetical diagram of the mechanisms of neurological damage due to homeostatic derangements of the Trx system caused by chemical toxins. Chemical stimuli (like methylglyoxal and nitrous oxide) inhibit TrxR, inhibit Trx reduction, or directly cause TXNIP to enter mitochondria. In the cytoplasm, TrxR cannot reduce Trx1, and TXNIP dissociates from the Trx1-TXNIP complex, causing cellular oxidative stress. Moreover, ASK1 separates from the Trx-ASK1 complex, resulting in apoptosis. In mitochondria, TXNIP dissociates from the Trx2-TXNIP complex and binds to NLRP3, releasing caspase-1 and -12 to cause apoptosis. At the same time, NF-κB undergoes nuclear translocation, releases inflammatory factors (TNF-α, IL-1 β, IL-6), activates p38, and ultimately causes apoptosis. The TXNIP-NLRP3 complex in mitochondria induces oxidative stress. ASK1 then separates from the Trx2-ASK1 complex, activating p38 and triggering apoptosis. Morphine phosphorylates ERK and Akt, activating the Trx1/CREB pathway, causing morphine addiction. Trx: thioredoxin; TrxR: Trx reductase; TXNIP: Trx-interacting protein; NLRP3: pyrin domain-containing-3; NF-κB: nuclear transcription factor-κB; ASK1: apoptotic signaling-regulated kinase 1; ERK: extracellular signal-regulated kinase; Akt: protein kinase B; CREB: cyclic adenosine monophosphate-responsive element-binding protein; TNF-α: tumor necrosis factor-α; IL-6: interleukin-6; IL-1β: interleukin-1β; ROS: reactive oxygen species.

TXNIP, a regulator of oxidative stress, is involved in cell proliferation, differentiation, and apoptosis. This protein is expressed in both the cytoplasm and mitochondria. It is the sole endogenous Trx-binding protein inhibiting Trx activity, thereby inducing oxidative stress and causing neurological disorders (e.g., dementia and cognitive deficit) [28]. TXNIP expression is upregulated in neurodegenerative disorders (e.g., AD) and cerebrovascular diseases (e.g., stroke) [5]. TXNIP overexpression has been observed in the hippocampus of transgenic mice with familial AD. The inhibition of TXNIP expression reduces the hippocampal levels of inflammatory factors such as interleukin-1β [29]. Under oxidative stress conditions, TXNIP dissociates from the TXNIP–Trx complex and binds to the nucleotide-binding domain, leucine-rich-containing family, and pyrin domain-containing-3 (NLRP3) inflammasome. This binding activates the NLRP3 inflammasome, activating caspase-1, which contributes to oxidative stress and leads to apoptosis and inflammation (Figure 3) [30].

## 3. Chemicals Damaging Nerves by Disrupting Trx System Homeostasis

### 3.1. Chemicals Damaging Nerves through Abnormal Trx Expression

#### 3.1.1. Morphine-Induced Overexpression of Trx Causes Nerve Damage

Opioids, such as morphine, are the most effective analgesics for the clinical treatment of chronic pain. However, their clinical use induces various side effects, including increased susceptibility to respiratory depression, withdrawal syndrome, and reward effects inherent to opioid use. Moreover, opioids do not eliminate the risk of relapse. Dopaminergic neurons in brain regions such as the ventral tegmental area (VTA), nucleus accumbens (NAc), and hippocampus have been implicated in morphine addiction, which interferes with the long-term plasticity of the brain [31]. Luo et al. [32] demonstrated that after treatment of SH-SY5Y cells with 10 µM of morphine for 24 h, Western blot showed elevated levels of Trx1. Morphine induced the expression of Trx1 in SH-SY5Y cells by activating opioid receptors; this induction resulted in hypermotility, reward effects, and withdrawal syndrome. The nerve growth factor triggers the binding of cyclic adenosine monophosphate (cAMP)-responsive element-binding protein (CREB) to the cAMP-responsive element of the Trx promoter and subsequently mediates the nuclear translocation of Trx. The nerve growth factor induces the expression of Trx1 through the extracellular signal-regulated kinase (ERK) and cyclic adenosine monophosphate-responsive element (CRE)/CREB pathways. Trx1, an essential element of the Trx promoter, is a downstream target of the phosphatidylinositol-3-kinase (PI3K)/protein kinase B (Akt) and ERK pathways. Morphine treatment increases the levels of Akt and ERK phosphorylation by inducing the translocation of Trx1 from the cytoplasm to the nucleus; consequently, the nuclear level of Trx1 is increased. By contrast, PI3K and ERK inhibitors reduce the level of Trx1. When SH-SY5Y cells were treated with a PI3K inhibitor (LY294002) or an ERK inhibitor (PD98059) for 30 min and then co-cultured with 10 µM morphine for 24 h, a morphine-induced increase in Trx1 expression was inhibited. In the nucleus, Trx1 promotes gene transcription by enhancing the interactions of nuclear transcription factor-κB (NF-κB) and CREB with DNA or coactivators (Figure 3). Evidence suggests that NF-κB and CREB play key roles in morphine addiction [33]. Therefore, Trx1 overexpression mediates morphine addiction. Long-term morphine addiction can directly inhibit the respiratory center and affect the central nervous system, causing cerebral ischemia and hypoxia [34]. Morphine addiction can induce compulsive drug-taking behavior and high rates of relapse. When withdrawing from morphine, there are withdrawal reactions such as severe anxiety and depression. Opioid receptor antagonists are commonly used to induce morphine withdrawal [35,36].

#### 3.1.2. Methamphetamine-Induced Inhibition of Trx Expression Causes Nerve Damage

Methamphetamine, a common psychostimulant, belongs to the family of phenylisopropylamine drugs. The misuse of methamphetamine can lead to oxidative stress, dopamine neuron toxicity, and neurological impairment. Prolonged methamphetamine abuse increases the risk of PD [37]. Yang et al. [38] revealed that methamphetamine induces apoptosis and autophagy in dopaminergic neurons through the endoplasmic reticulum stress pathway mediated by CCAAT/enhancer-binding protein homologous protein (CHOP). Methamphetamine exposure increases glutamatergic transmission (N-methyl-D-aspartate receptor subunit 2B and glutamate) and autophagy in mice, induces endoplasmic reticulum stress, upregulates the expression of CHOP, glucose-regulated protein 78 antigen, and B-cell lymphoma-2 (Bcl-2)-associated X protein (Bax; proapoptotic factor) in the VTA and NAc, and downregulates the expression of Bcl-2 (antiapoptotic factor) and caspase-12. Furthermore, methamphetamine exposure leads to a marked reduction in the expression level of Trx1 in the VTA and NAc of mice after intraperitoneal injection of methamphetamine (2.5 mg/kg) for 8 days. Similarly, this is caused by increased glutamatergic transmission in VTA and NAc by methamphetamine exposure, through activation of the CHOP-mediated endoplasmic reticulum stress signaling pathway and mitochondrial apoptosis, resulting in brain damage, rewarding effects, and central nervous system injury. Overexpression of Trx1 using transgenic technology can inhibit the methamphetamine-induced increase in glutamatergic transmission and can reduce endoplasmic reticulum stress, thereby mitigating methamphetamine-induced nerve damage.

### 3.2. Chemicals Damaging Nerves through the Inhibition of TrxR Activity

#### 3.2.1. Methylglyoxal Inhibits TrxR-Induced Nerve Damage

Methylglyoxal, a metabolite found in many organisms, spontaneously reacts with biopolymers, which are rearranged to form stable advanced glycosylation end products. These end products are involved in the development of aging-related diseases such as neurodegenerative disorders, cancer, and diabetes mellitus (DM) [39].

The neuronal cell line HT22 serves as an excellent model for studying neurodegenerative disorders. Dafre et al. [40] observed increased glycosylation, inactivation of TrxR1 in the nucleus and cytoplasm, and inhibition of Trx1 expression in methylglyoxal-treated HT22 cells. The content of Trx1 in HT22 cells was decreased after treatment with 0.75 mM methylglyoxal for 24 h. Treated with the same concentration for 30 min, the content of TrxR1 decreased; however, no TrxR1 glycosylation was observed in untreated cells. There is a direct effect of methylglyoxal on TrxR1, resulting in inactivation. It was also observed that TrxR2, which is expressed in mitochondria, was reduced by 25% when HT22 cells were treated with 0.75 mM methylglyoxal for 8 h. These findings suggest that Trx and TrxR are key targets for the toxic effects of methylglyoxal. Schmitz et al. [41] demonstrated that the toxic effects of methylglyoxal on hippocampal brain slices and HT22 cells were exacerbated when TrxR activity was suppressed using 2-acetylamino-3-[4-(2-acetylamino-2-carboxyethylsulf anylthiocarbonylamino)phenylthiocarbamoylsulfanyl]propionic acid (2-AAPA) and auranofin. Treatment of SH-SY5Y cells with 625 μM methylglyoxal alone for 24 h did not cause cell death, whereas pretreatment of SH-SY5Y cells with either 10 μM 2-AAPA or 0.5 μM auranofin for 30 min, followed by treatment with 625 μM methylglyoxal for 24 h, resulted in a decrease in cell viability by about 20%. As a result, TrxR inhibition increases the toxicity of methylglyoxal, exacerbating oxidative stress in nerve cells and acting in concert with each other. Furthermore, elevated levels of methylglyoxal have been detected in the plasma of patients with DM; thus, methylglyoxal may be involved in the onset and progression of DPN [42]. The increased intracellular accumulation of methylglyoxal reduces the activity and expression of Trx2 in the mitochondria, leading to the dissociation of Trx2 and ASK1 and the activation of the ASK1/p38 mitogen-activated protein kinase (MAPK) pathway (Figure 3). These events increase the levels of mitochondrial ROS, leading to mitochondrial dysfunction, impaired insulin synthesis and secretion, and β-cell apoptosis [43].

#### 3.2.2. Metals and Metalloids Inhibit Trx and TrxR, Inducing Nerve Damage

Metal or metal-like ions, such as gold, silver, platinum, mercury, and arsenic, are typically soft acids. The deprotonated forms of the selenol of Sec in TrxR can function as a soft base, exhibiting a high affinity for soft acids. Partial deprotonation of thiols in the N-terminal Cys-Val-Asn-Val-Gly-Cys (CVNVGC) active site transforms the active site into a site targeting soft acids. The interactions between soft bases and acids inhibit TrxR activity and convert Trx-(SH)_2_ to Trx-S_2_ (Figure 2), leading to DNA damage, elevated ROS levels, cell cycle changes, enzyme activity loss, and apoptosis. Compounds of gold—both Au(I) and Au(III) states—exhibit cytotoxicity. These compounds can selectively inhibit mammalian TrxR activity, block Trx reduction, oxidize Trx, and generate ROS [44]. Wang et al. [45] observed that treatment of SH-SY5Y cells with 10 µM cadmium chloride for 12 or 24 h resulted in a decrease in TrxR1 levels. Cadmium markedly suppresses endogenous TrxR1 expression and enzyme activity in neurons, reduces cell viability, and increases intracellular ROS levels and apoptosis, thereby impairing the differentiation of adult hippocampal neurons. These findings suggest that TrxR1 is a key target for cadmium [46]. A recent study claims that the expression of the TXNIP and NLRP3 proteins was upregulated after SH-SY5Y cells were treated with 10 μM cadmium chloride for 24 h. Cadmium can also induce endoplasmic reticulum stress and excess ROS, leading to neuroinflammation [47].

Auranofin, a gold-containing compound, serves as a mitochondrial toxicant. It substantially increases the severity of mechanical anomalous pain and the level of mechanical nociceptive sensitization in painful peripheral neuropathy [48]. Yumnamcha et al. [49] demonstrated that auranofin specifically inhibits TrxR1 and TrxR2, leading to mitochondrial dysfunction in retinal pigment epithelial cells, mitochondrial autophagy, and inflammatory pyroptosis. The aforementioned findings suggest that auranofin’s interference with the Trx/TrxR pathway contributes to cellular oxidative stress, mitochondrial dysfunction, altered mitochondrial autophagy, lysosomal damage, and proinflammatory cell death in retinal neurodegenerative disorders. Therefore, maintaining the homeostasis of the Trx/TrxR redox system may prevent the progression of various neurodegenerative disorders.

### 3.3. Chemicals Damaging Nerves through the Activation of TXNIP

#### 3.3.1. Streptozotocin-Induced Activation of TXNIP Causes Nerve Damage

Streptozotocin is a classical compound that induces DM and DPN [50]. Gao et al. [51] found that the expression of TXNIP is upregulated in the dorsal root ganglion of rats with streptozotocin-induced DM (55 mg/kg for 3 days). The researchers indicated that TXNIP binds to Trx and inhibits its function and expression, enhancing NLRP3 inflammasome activation and inducing caspase-1 activation and inflammatory factor (tumor necrosis factor-α (TNF-α)) release. These events lead to apoptosis in pancreatic β-cells and abnormal nerve conduction through the induction of oxidative stress and the inflammatory response, resulting in DPN [4].

#### 3.3.2. Nitrous Oxide-Induced Activation of TXNIP Causes Nerve Damage

Nitrous oxide (laughing gas) is a colorless, nonirritating compound [52]. Its abuse has become a global problem. This gas is commonly used to induce anesthesia during outpatient dental procedures. However, recently, nitrous oxide has become popular among teenagers, who use it for recreational purposes [53]. Prolonged nitrous oxide abuse causes neurological damage, leading to myeloneuropathy [54] and motor axonal neuropathy [55]. cAMP is a secondary messenger essential for maintaining neuronal growth and orchestrating successful axonal regeneration. This compound phosphorylates CREB, which subsequently upregulates arginase 1 expression, increases polyamine production, and stimulates axon development. CREB regulates the body’s responses to neurotrophic factors, such as brain-derived growth factors [56]. Liu et al. [57] demonstrated that nitrous oxide (70% nitrous oxide, 5% carbon dioxide, and 25% oxygen for 6 h) promotes the release of inflammatory factors by activating the TXNIP/NLRP3 pathway. This activation results in the abnormal localization of NF-κB in the nucleus, which leads to the excessive release of inflammatory factors (TNF-α, interleukin-6 (IL-6), and interleukin-1β (IL-1β)). This cascade activates p38 MAPK, thereby causing hippocampal neuronal damage (Figure 3).

## 4. Regulation of Trx System Homeostasis Mitigates Nerve Damage

### 4.1. Upregulation of Trx Transcription and Expression Mitigates Nerve Damage

#### 4.1.1. Sulforaphane-Induced Expression of Trx Mitigates Nerve Damage

Sulforaphane, an aliphatic isothiocyanate, exhibits diverse biological activities, including antioxidative, anti-inflammatory, antitumor, antiangiogenic, and brain health-protective activities. Long-term diabetes increases the risk of diabetic retinopathy, in which Trx plays a key role [58]. Tang et al. [59] found that sulforaphane (0.5 μM for 24 h) upregulates the expression of Trx1 in the primary hippocampal neuron cells of Tubby mutant mice. In contrast to treatment with lysergic sulfanilamide alone, 4-week combination treatment with sulforaphane and PX12—an exogenous inhibitor of Trx1—markedly increases the rate of apoptosis and upregulates the expression of CHOP, Bax, phosphorylated c-Jun N-terminal kinase (JNK), and caspase-12. This is accompanied by the downregulation of Bcl-2 expression. Both the endoplasmic reticulum and mitochondria have damage similar to that observed in diabetic mice. These findings implicate Trx1 in the sulforaphane-mediated prevention of neuronal apoptosis in diabetic mice.

The Ras/Raf/ERK (MAPK) pathway, which is essential for intra- and extracellular communication, serves as a central effector in signaling cell differentiation during the normal growth and development of various tissues. This pathway regulates basic cellular functions such as cell survival, growth, and differentiation. It is necessary for the survival and maintenance of many neuronal cell populations [60]. Kong et al. [61] found that the intracardiac injection of sulforaphane (25 mg/kg for 1 day) had effects on the retinal levels of Trx in mice after 72 h of light exposure. Sulforaphane decelerates retinal degeneration by upregulating the expression of nuclear factor erythroid 2-related factor 2 and Trx in retinal nerves through the activation of the Ras/Raf1/ERK pathway, suppressing the release of cytochrome C and the activity of calpain I, regulating the rate of apoptosis, and protecting photoreceptor cells against photodamage. Sulforaphane activates ERK, which, in turn, induces the transactivation of the Ras and MAPK cascades and upregulates the expression of Trx, thereby preventing the downregulation of tubule-associated antioxidants and the death of cells.

#### 4.1.2. N-Acetylcysteine-Induced Expression of Trx Mitigates Nerve Damage

N-acetylcysteine, a synthetic derivative of endogenous L-cysteine, has been used for the management of neurodegenerative disorders, including spinal cerebellar ataxia, PD, delayed dyskinesia, and Unverricht–Lundborg-type myoclonic epilepsy. It is also used as adjunctive therapy for various diseases, such as multiple sclerosis, amyotrophic lateral sclerosis, and AD [62]. In starved neuronal cells (SH-SY5Y), treatment with 2.5 mM N-acetylcysteine for 24 h prevents TrxR1 knockout-mediated protein hydrolysis dysfunction, reduces protein aggregation, and enhances cell survival [63]. Li et al. [64] found that in neurodegenerative disorders caused by type 1 DM, a combination of N-acetylcysteine and insulin considerably increases Trx1 and Trx2 levels compared with insulin alone. This combination enhances the body’s antioxidative activity, mitigating oxidative damage in the brain. This discovery offers new insights for managing type 1 DM and its complications.

### 4.2. Selenium Supplementation-Induced Enhancement of TrxR1 Activity Mitigates Nerve Damage

Selenocysteine is present in at least 25 human selenoproteins that are involved in a wide range of biological functions. The active site of TrxR, a selenoprotein, contains selenocysteine residues [65]. Selenium is a key element that exhibits antioxidative and neuroprotective activities in various models of toxicity. It plays a central role in preventing and regulating a diverse range of diseases, such as cancer, diabetes, AD, psychiatric disorders, cardiovascular disease, fertility impairment, inflammation, and infections [66]. Wang et al. [46] found that selenium supplementation substantially reduces cadmium cytotoxicity in neuronal cells (SH-SY5Y) by enhancing TrxR1 activity and increasing TrxR1 levels in these cells. These changes exert antioxidative effects, reducing ROS production and apoptosis and mitigating cadmium-induced apoptosis. The researchers also found that, compared with 20 μM selenium selenite, 5 μM selenium selenite markedly enhanced TrxR1 activity in neuronal cells. This finding suggests that exposure to low concentrations of selenium can protect the nervous system. Thus, selenium may be applied in the prevention and treatment of neurodegenerative disorders, such as AD and PD, by enhancing TrxR1 activity and strengthening antioxidative effects.

### 4.3. Inhibition of TXNIP Mitigates Nerve Damage

#### 4.3.1. Vitamin D_3_-Induced Inhibition of TXNIP Mitigates Nerve Damage

Vitamin D_3_ (cholecalciferol) serves as a pleiotropic neurosteroid in the brain. Its active form, 1,25(OH)_2_D_3_, also serves as a neurosteroid, acting as both a hormone and a paracrine/autocrine agent in the brain [67]. Epidemiologic and clinical evidence suggests that a higher level of vitamin D in serum is associated with higher cognitive performance; by contrast, a low level of vitamin D is associated with AD development [68]. Lu et al. [69] reported that vitamin D_3_ (233.3 U/kg body weight/week for 6 months) alleviates retinal inflammation by reducing ROS production (in the retinal vascular endothelium of rats), the activity of the TXNIP/NLRP3 pathway, the Bax/Bcl-2 ratio, retinal apoptosis, and retinal tissue damage, thereby ameliorating DM-related retinal damage. Gao et al. [51] revealed that peripheral nerve conduction velocity in patients with DM is associated with TXNIP. Vitamin D_3_ can inhibit inflammation and reduce neuronal apoptosis, thereby preserving peripheral nerve function in patients with DM. The downregulation of TXNIP expression mitigates oxidative stress in patients with DPN.

#### 4.3.2. Resveratrol-Induced Inhibition of TXNIP Expression Mitigates Nerve Damage

Resveratrol, which is derived from grape seeds, is a polyphenolic organic compound. It is easily absorbed by the gastrointestinal tract, metabolized, and excreted through the urine and feces. Han et al. [70] established an animal model of DM by feeding SD rats a high-fat, high-sugar diet and by injecting (laparoscopically) the animals with 1% streptozotocin. Then, these animals were treated with resveratrol (10 mg/kg intraperitoneally for 6 weeks). As a TXNIP inhibitor, resveratrol decreased the synthesis of NLRP3 and suppressed TXNIP expression. This increased sciatic nerve conduction velocity, mitigated pathological changes in sciatic nerve myelin, and lessened symptoms associated with sciatic nerve conduction injury. Notably, miR-146a-5p, the most abundant miRNA in exosomes [71], is involved in cell proliferation [72]. Fan et al. [73] implicated microglia-specific miR-146a-5p in the development of depression, revealing a new therapeutic target for this condition. Hu et al. [74] indicated that resveratrol ameliorates DM-induced cognitive impairment through the miR-146a-5p/TXNIP axis. In patients with DPN, miR-146a-5p increases nerve conduction velocity, mitigates morphological damage, and reduces sciatic nerve demyelination. TXNIP may be a target of miR-146a-5p because miR-146a-5p overexpression considerably downregulates the expression of TXNIP. Resveratrol upregulates the expression of miR-146a-5p, thereby inhibiting TXNIP expression and alleviating the cognitive impairment associated with diabetic encephalopathy. Together, these findings highlight the potential of resveratrol as an inhibitor of TXNIP expression.

## 5. Summary and Outlook

Several studies have revealed the crucial role of the Trx system in the regulation of nerve injury. Trx and TrxR serve as key targets in chemical nerve injury. In addition to these targets, TXNIP—an endogenous Trx-binding inhibitory protein—was explored in this review. We mentioned that the Trx system plays a role in the maintenance of redox homeostasis in the central and peripheral nervous systems, which is altered in neurodegenerative disorders. Studies on postmortem brains from different neurodegenerative disorders have revealed a differential modulating pattern in these disorders by the Trx system. Reduced levels of selenium and Trx1 in the brains of patients with AD are linked to the oxidation of Trx1 caused by Aβ peptides, which separates Trx1 from ASK1 and initiates apoptosis. In the brains of individuals after death, TrxR1 levels may rise compensatorily. In the brains of patients with Parkinson’s disease, the neurotoxic MPP^+^ is generated, which results in lowered levels of both Trx1 and TrxR2. The brains of patients with Parkinson’s disease lack the antioxidant protein DJ-1, which prevents it from co-immunoprecipitating with free ASK1, which ultimately triggers apoptosis through the caspase-12 pathway. Patients with Huntington’s disease have diminished TrxR1 and Trx1 levels due to dysregulated glutathione (GSH) redox. TXNIP is high in brain stroke patients, and the ASK1/p38 pathway is activated. TXNIP inhibitors cause thromboembolic stroke prevention [6].

We found that chemicals such as morphine, metals, and methylglyoxal induce nerve injury by inhibiting Trx and TrxR expression and upregulating TXNIP expression. Of course, the chemicals that target the Trx system to cause chemical nerve damage go far beyond what we have discussed. The anti-cancer drug paclitaxel causes peripheral neuropathy with neuropathic pain. Research says it induces sciatic nerve pain in rats by inhibiting TrxR2 [48]. Botulinum neurotoxins inhibit Trx/TrxR, leading to peripheral nerve paralysis [75]. A high-glucose environment may also induce TXNIP, which causes oxidative stress in microglia and diabetic neuroinflammation [76]. Homeostatic imbalances in the Trx system caused by chemical nerve injury are mediated primarily through the PI3K/Akt, ERK, ASK1/p38 MAPK, TXNIP/NLRP3, or cAMP/CREB pathways. The results of earlier research that were referenced in this study were combined by us. We proposed the hypothesis that, when chemically stimulated, in the cytoplasm, TrxR cannot reduce Trx1, and TXNIP dissociates from the Trx1-TXNIP complex, causing cellular oxidative stress. Moreover, ASK1 separates from the Trx-ASK1 complex, resulting in apoptosis. When cells are stimulated by nitrous oxide, TXNIP in mitochondria dissociates from the Trx2-TXNIP complex and binds to NLRP3, releasing caspase-1 and -12 to cause apoptosis. At the same time, NF-κB undergoes nuclear translocation, releases inflammatory factors (TNF-α, IL-1 β, IL-6), activates p38, and ultimately causes apoptosis. When stimulated by methylglyoxal, the TXNIP-NLRP3 complex in mitochondria induces oxidative stress. ASK1 then separates from the Trx2-ASK1 complex, activating p38 and triggering apoptosis. Chemical nerve damage is the result of this (Figure 3). Researchers have identified multiple therapeutic agents for regulating these pathways—for example, sulforaphane, resveratrol, vitamin D_3_, and selenium. Research has claimed that docosahexaenoic acid, as an indirect antioxidant, enhances the antioxidant capacity of the Trx system in nerve cells and may have a big breakthrough in neuroprotective research on AD [77]. The upregulation of endogenous Trx1 and Trx2 expression and the administration of exogenous Trx1 inducers can confer neuroprotective effects against AD through the activation of prosurvival pathways (PI3K/Akt and ERK pathways) and the suppression of oxidative stress, inflammation (TXNIP, NLRP3, and NF-κB), and apoptosis (ASK1/JNK/p38 MAPK and the caspase-1 and -12 pathways) [78]. Therefore, maintaining the homeostasis of the Trx system by upregulating Trx1 and Trx2 expression, enhancing TrxR activity, and inhibiting TXNIP may protect against chemical nerve injury. Evidence suggests that both Trx1 suppression and overexpression can lead to nerve damage, and the benefits of Trx1 are not absolute. Morphine phosphorylates ERK and Akt, activating the Trx1/CREB pathway, causing morphine addiction (Figure 3). These findings highlight the need for a practical orientation in targeting Trx1 and Trx2 for the treatment of chemical nerve injury. In conclusion, the precise molecular mechanisms underlying the homeostatic imbalance of the Trx system in chemical nerve injury remain unclear. Strategies for regulating the Trx system to treat nerve damage are in the experimental stage. In the clinical practice of the therapy of chemical nerve injury, Trx1,2 agonists, TrxR agonists, and TXNIP inhibitors may be developed. Researchers can also consider sulforaphane, resveratrol, selenium supplementation, and other substances mentioned that can modulate the homeostasis of the Trx system. Future studies should focus on the clinical applications of these strategies, facilitating the development of Trx system-targeting drugs for the treatment of chemical nerve injury.

## Data Availability

Not applicable.

## Abbreviations

2-AAPA	2-acetylamino-3-[4-(2-acetylamino-2-carboxyethylsulf anylthiocarbonylamino)phenylthiocarbamoylsulfanyl]propionic acid
AD	Alzheimer’s disease
Akt	Protein kinase B
ASK1	Apoptotic signaling-regulated kinase 1
ATP	Adenosine triphosphate
Bax	B-cell lymphoma-2-associated X protein
Bcl-2	B-cell lymphoma-2
cAMP	Cyclic adenosine monophosphate
CHOP	CCAAT/enhancer-binding protein homologous protein
CRE	Cyclic adenosine monophosphate-responsive element
CREB	Cyclic adenosine monophosphate-responsive element-binding protein
CVNVGC	-Cys-Val-Asn-Val-Gly-Cys-
DM	Diabetes mellitus
DPN	Diabetic peripheral neuropathy
ERK	Extracellular signal-regulated kinase
GSH	Glutathione
IL-1β	Interleukin-1β
IL-6	Interleukin-6
JNK	Phosphorylated c-Jun N-terminal kinase
MAPK	Mitogen-activated protein kinase
MPP^+^	1-methyl-4-phenylpyridinium ion
NAc	Nucleus accumbens
NADPH	Nicotinamide adenine dinucleotide phosphate
NF-κB	Nuclear transcription factor-κB
NLRP3	Pyrin domain-containing-3
PD	Parkinson’s disease
PI3K	Phosphatidylinositol-3-kinase
ROS	Reactive oxygen species
TNF-α	Tumor necrosis factor-α
Trx	Thioredoxin
TrxR	Trx reductase
TXNIP	Trx-interacting protein
VTA	Ventral tegmental area
